# Reply to: The case for standardizing gene nomenclature in vertebrates

**DOI:** 10.1038/s41586-022-05634-9

**Published:** 2023-02-15

**Authors:** Constantina Theofanopoulou, Erich D. Jarvis

**Affiliations:** 1 Laboratory of Neurogenetics of Language, Rockefeller University, New York, NY USA; 2 Hunter College, City University of New York, New York, NY USA; 3 Howard Hughes Medical Institute, Chevy Chase, MD USA

**Keywords:** Phylogenetics, Evolutionary genetics, Phylogeny, Genomics

replying to F. M. McCarthy et al. *Nature* 10.1038/s41586-022-05633-w (2023)

Here we reply to points raised by McCarthy et al. in the accompanying Comment^1^ concerning our proposal^2^ for an evolution-based and universal vertebrate nomenclature for the oxytocin and vasotocin ligand and receptor families, and the principles considered for homology-based gene nomenclatures. We strengthen our claims with additional evidence and propose evidence-based criteria for homologous gene nomenclature, in the following order of reliability: synteny, phylogenetic inference, sequence identity and gene function. We believe that the time is ripe for gene nomenclature committees and initiatives generating high-quality assemblies to join forces in a universal gene nomenclature committee.

Our proposed universal gene nomenclature (that is, naming) for the oxytocin and vasotocin ligands and receptors^2^ was based on several criteria, including gene synteny, phylogeny, identity and function, and provides a case study that is applicable across gene families. McCarthy et al.^1^ argue that a standardized system of nomenclature already exists, “first established in vertebrates 30 years ago”, and that only minor changes are needed in this gene family, with a focus on tradition, name stability, phylogeny, identity and gene function, and with the order of priority of evidence determined on a case-by-case basis. We disagree with both of these claims, especially because determining gene orthology was not fully possible until the recent availability of high-quality genomes. Below, we discuss the principles that we suggest should be applied across gene families and future initiatives. In the Supplementary Information, we respond to the gene-specific claims made by McCarthy et al.^1^.

In our study^2^, for each of the oxytocin and vasotocin ligands and receptors, we listed two to six commonly used aliases (Table 1 in Theofanopoulou et al.^2^). Many of these reflect incorrect orthologies or paralogies, indicating that there was not a universally used standard before our study, nor one that sufficiently portrayed gene orthology. We view the vertebrate-wide gene nomenclature that McCarthy et al.^1^ present as “approved” in their Table 1 as newly proposed. They adopted the most common gene names for mammals, revised some on the basis of our study and others, and applied them to all other vertebrates where possible (Supplementary Note 1). None of the other aliases were listed, which makes the translation of findings across species and the literature difficult. Furthermore, in their newly proposed nomenclature, tradition overrides orthology and paralogy. For example, they maintain very different names for the genes oxytocin and vasotocin that do not echo their paralogy (that is, oxytocin and arginine vasopressin); and for species that do not have the arginine amino acid, they change the name to another alias (vasopressin), but still abbreviate it *to AVP*. We think that allowing tradition and stability to override naming rules of orthology and paralogy could lead to confusion.

However, we believe it is possible to consider both tradition and orthology/paralogy. For example, because vasotocin is the evolutionarily older gene, with oxytocin resulting from a local duplication of it^2^, if we were strict with evolutionary naming, we would have renamed vasotocin to ‘vasotocin 1’ and oxytocin to ‘vasotocin 2’. But to conserve some continuity with traditional use, we proposed the already used ‘vasotocin’ for vasopressin, to mirror the ending of ‘oxytocin’. In forming this proposal, we consulted with experts, whom we acknowledged^2^, and with the leaders of the Ensembl annotation team.

Valuing accuracy over tradition comes with some downsides. Perhaps the greatest would be the effort required to ensure continuity between previous publications and annotated genomes with the new nomenclature. To mitigate this, we suggest a translation table from old to revised gene names (for example, Table 1 in Theofanopoulou et al.^2^), which would be available in platforms like the National Center for Biotechnology Information. Current committees already use such tables, but their practices of establishing nomenclature changes are either different than the ones we propose or not consistent with each other (Supplementary Notes 2–4).

McCarthy et al.^1^ also criticize our proposed two-letter symbols for oxytocin and vasotocin (*OT* and *VT*), in that they give broader results in a literature search compared to three-letter symbols (such as *OXT* and *AVP*). We agree and further argue that three-letter symbols could still reflect an evolution-based nomenclature; for example, *OTC* (oxytocin) and *VTC* (vasotocin). We also suggest that gene-symbol consistency across species should be adopted in their letter capitalization. The landscape at present, in which only some mammalian and avian gene symbols are upper case, mouse and rat symbols are lower case except for an initial upper-case letter and amphibian and fish species are all lower case, does not depict the real orthology of these genes, and perpetuates anthropocentric practices. In our universal nomenclature proposal^2^, we suggest that gene symbols should be upper case across species.

We agree with McCarthy et al.^1^ that for name revisions, the benefits should outweigh the risks. We are guided by the belief that “names have a powerful influence on the experiments we do and the way in which we think”^3^, and hence it is important that names do not give rise to false expectations. For example, the binding of oxytocin to the ‘vasopressin’ receptors has been often identified as surprising—something that could be avoided with names that reflect their common origin (-tocin). This knowledge will also be useful for medicine, so that physicians are more aware of drug interactions between the two receptor families. Similarly, in other gene families, McCarthy et al.^1^ endorse a nomenclature that differs in orthologous genes with a different function across species. For example, the *CSAD* gene is named ‘cysteine sulfinic acid decarboxylase’ in all species except chickens, in which it is called ‘cysteine acid decarboxylase’. If sequence and/or function changes were routinely used to change gene names, then nearly all orthologous genes would have different names across species.

McCarthy et al.^1^ decided not to suggest blanket ‘rules’ about which factors should be weighed more heavily than others, as each case will differ depending on the context. In our experience, not all evidence has equal weight. For example, McCarthy et al.^1^ did not accept our nomenclature in part due to the lack of sequence-identity resolution (Basic Local Alignment Search Tool (BLAST) analyses). However, sequence-identity percentages do not always provide a solid basis for gene nomenclature, because orthologous syntenic genes can misleadingly have higher sequence identity with a paralogous gene (Supplementary Table 12 in Theofanopoulou et al.^2^). In addition, McCarthy et al.^1^ presented an amino acid phylogeny as not being conclusive enough for some of our interpretations. However, we showed^2^ that amino acid phylogenies have low bootstrap support on some branches, whereas exonic nucleotide phylogenies yielded a higher resolution that supports our conclusions (Fig. 4 in Theofanopoulou et al.^2^). With more high-quality genome assemblies generated by the Vertebrate Genomes Project (VGP) since our original publication, we ran a new exonic phylogeny that even more strongly supports our conclusions (Fig. 1 and Supplementary Notes 3 and 4).

**Fig. 1:**
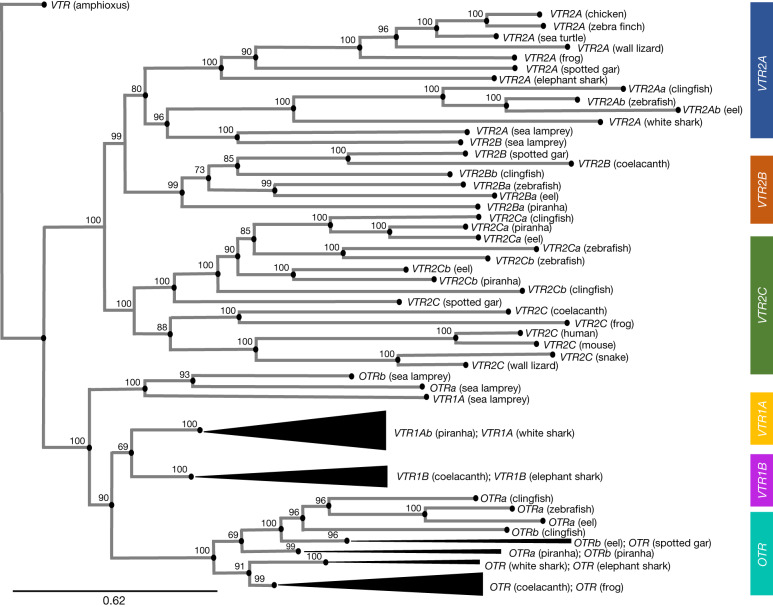
Family tree for genes that encode the oxytocin and vasopressin receptors. Tree topology inferred with the phylogenetic maximum likelihood method on an exon nucleotide alignment (MAFFT), with 1,000 non-parametric bootstrap replicates (IQ-TREE). Bootstrap values are shown as percentages at the branch points. The tree is rooted with the *VTR* gene in amphioxus. The gene names of the current accessions (see Table 1 in Theofanopoulou et al.^2^ and Supplementary Tables 4a–e in Theofanopoulou et al.^2^ for a full list of synonyms) were written over according to our revised synteny- and phylogeny-based orthology. All sequences used, FASTA alignment and Newick tree files can be accessed here at https://github.com/constantinatheo/universalnomenclature/. Scale bar, 0.62 substitutions. For a discussion on interchanging *VTR2A* and *VTR2C* naming, see Supplementary Note 3.

We find^2,4^ that synteny-based approaches in most cases give the best resolution for gene orthologies and paralogies, and hence for gene nomenclature. Wherever available, we propose using chromosome-scale genomes that are highly contiguous and have a high base-call accuracy^2^. When synteny is not clear, we suggest that priority is given to nucleotide phylogenetic inference with the same prerequisites for genome quality. In Extended Data Fig. 1 and Supplementary Note 5, we provide specific suggestions and caveats with regard to our recommended practices for synteny and phylogenetic analyses. We propose that a combination of synteny and highly supported phylogeny is the backbone of a universal gene nomenclature.

According to the guidelines for human gene nomenclature^5^, initiatives that aim to revise a nomenclature when the old one is “misleading…are welcomed”. We agree with this practice. However, we believe that the process that is used to approve those revisions should take a different approach to the ones proposed by McCarthy et al.^1^ We do not think that journal editors should require “scientists to consistently use approved nomenclature”^1^ by a limited committee. Rather, we believe that they should allow new uses in the light of new evidence (see checklist in Extended Data Fig. 1).

Moreover, the current nomenclature committees represent nomenclature focused on only 0.01% of the 70,000 extant vertebrates, with genome assemblies that were much more fragmented, and with traditions that we think need reconsideration. Although several authors of the accompanying Comment by McCarthy et al.^1^ are part of a recently formed Vertebrate Gene Nomenclature Committee (VGNC), in their database (https://vertebrate.genenames.org/) at the time of writing (19 November 2022) there is no inclusion of gene aliases used in the literature (versus Table 1 in Theofanopoulou et al.^2^).

The high-quality genomes generated by the VGP (https://vertebrategenomesproject.org/) and related initiatives such as the Earth BioGenome Project (https://www.earthbiogenome.org/)^6,7^ are greatly improving the identification of gene orthology and thereby gene annotation, bringing about an opportunity to establish a universal nomenclature for most genes. Our experience in these initiatives is that existing gene annotation and nomenclature bodies are not yet coordinated or consistent in their approaches. We envisage a universal gene nomenclature committee that involves scientists working on sequencing, assembly, annotation, phylogeny and genome evolution, as well as on the respective lineages and genes for all life.

One possible organizing principle would be to create one committee per major lineage (for example, cyclostomes), group these as subcommittees under one larger committee (for example, all vertebrate species), group all of them under a committee for all species of one of the animal kingdoms (for example, eukaryotic species) and then group all of them under all life. We believe that such an effort would be likely to require changes both to infrastructure (for example, committees and publication policies) and to the way systems operate (for example, high-quality genomes, synteny and phylogenetics).

## Reporting summary

Further information on research design is available in the Nature Portfolio Reporting Summary linked to this article.

## Supplementary information

Supplementary Notes This document contains detailed observations on the following topics: Supplementary Note 1: standardization biases; Supplementary Note 2: factors in favour of or against nomenclature change; Supplementary Note 3: *VTR2A* and *VTR2C*: which is most likely the oldest and why; Supplementary Note 4: is *VTR2Ab* in teleost fish a *VTR2A* duplicate?; and Supplementary Note 5: suggested checklist for evolution-based gene nomenclatures.

Reporting Summary

Supplementary Tables This file contains information on the species’ genomes (Supplementary Table 1) and specific genes analysed (Supplementary Table 2) for the exonic phylogeny in Fig. 1.

## Figures and Tables

**Extended Data Fig. 1:**
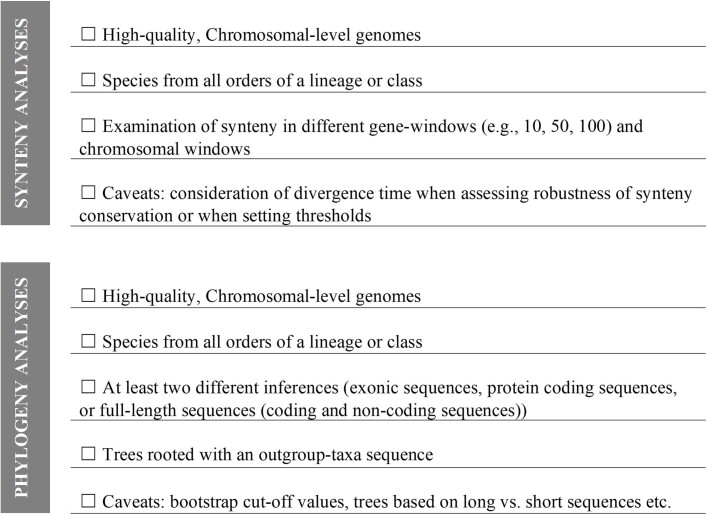
Checklist and caveats. Suggested checklist and caveats to be considered for synteny (top) and phylogeny (bottom) evidence used to propose gene nomenclature.

## Data Availability

All of the data used can be found in the Supplementary Notes and in the following repository: https://github.com/constantinatheo/universalnomenclature.

## Code availability

All the code used in this study can be found in the following repository: https://github.com/constantinatheo/universalnomenclature.
