# Supplementary material for: Reply to: The case for standardizing gene nomenclature in vertebrates
Source: Nature. 2023 Feb 15;614(7948):E33–6. doi: 10.1038/s41586-022-05634-9 (PMC9931572; doi:10.1038/s41586-022-05634-9)
Supplement: Supplementary file 1 — This document contains detailed observations on the following topics: Supplementary Note 1: standardization biases; Supplementary Note 2: factors in favour of or against nomenclature change; Supplementary Note 3: VTR2A and VTR2C: which is most likely the oldest and why; Supplementary Note 4: is VTR2Ab in teleost fish a VTR2A duplicate?; and Supplementary Note 5: suggested checklist for evolution-based gene nomenclatures. [file 41586_2022_5634_MOESM1_ESM.docx]

**Reply to: The Case for Standardising Gene Nomenclature in Vertebrates**

**Constantina Theofanopoulou*^1,2^ and Erich D. Jarvis*^1,2,3^**

**^1^**Laboratory of Neurogenetics of Language, Rockefeller University, New York, USA

**^2^** Hunter College, City University of New York, New York, USA

**^3^** Howard Hughes Medical Institute, Chevy Chase, Maryland, USA

*correspondence to: [ktheofanop@rockefeller.edu](mailto:ktheofanop@rockefeller.edu) and [ejarvis@mail.rockefeller.edu](mailto:ejarvis@mail.rockefeller.edu)

**Supplementary Notes**

In this supplement, we focus on issues raised by *McCarthy et al.*^1^ in their supplement on our revisions of the oxytocin and vasotocin ligand and receptor genes in *Theofanopoulou et al*^2^. In the main text, we already addressed the issues of consulting with the research community, working with NCBI, and 3+ vs. 2 letter gene symbol abbreviations.

**Supplementary Note 1**

**Standardization biases**

*McCarthy et al.*^1^ argue that commonly used terms should take precedence when considering nomenclature change. They challenged our claim^2^ that vasotocin is commonly used, where they performed a PubMed search showing that vasotocin returns 2,581 results and vasopressin 48,281. However, we said vasotocin is commonly used for the gene name in non-mammalian species and vasopressin in mammals. Here we conducted a more refined PubMed search (as of 9/12/2022), and find that of 48,570 results for vasopressin, 43,956 (90.5%) are associated with ‘mammal’ and only 327 (0.67%) with ‘bird’ (same for avian). In contrast, we find that of 2,592 results for vasotocin, 1,336 (51.5%) are associated with ‘mammal’ and 491 (18.9%) with ‘bird’ (similar for avian). This indicates a mammalian bias in the number of publications in the literature and the name revision proposal presented by *McCarthy et al.*^1^, as their approach substitutes a gene name commonly used in mammals over a gene name commonly used in all other vertebrates and many invertebrates, spanning over a million more species. Overall the “minor” revisions that *McCarthy et al.*^1^ proposed is to take the name most commonly used for mammalian species (that includes human) and propagate them to all other species. While some scientists are comfortable with such a mammalian bias, the problem in this case is that it takes a non-orthology-based and misleading nomenclature (e.g., arginine vasopressin and oxytocin) and overrides a sound orthology-based nomenclature (e.g., vasotocin and oxytocin) across the animal kingdom. We think the reverse is more conducive to scientific progress.

**Supplementary Note 2**

**Factors in favor or against nomenclature change**

*McCarthy et al.*^1^ put forward a table of six factors to consider in favor or against changes to an existing nomenclature, based on whether: 1) The existing nomenclature is incorrect or misleading; 2) The existing nomenclature is causing confusion; 3) The existing nomenclature is not being widely used; 4) A change in the existing nomenclature would significantly improve communication about a gene; 5) In the existing nomenclature there is not a unique or good search term; and 6) The existing nomenclature is not reflecting evolutionary relationships. They argue that with the exception of lack of evolutionary relationship reflection in the names between oxytocin and arginine vasopressin, all the five remaining factors are already satisfied in the current commonly used nomenclature, with only minor updates. We disagree. The current system for this gene family of ligands and receptors is confusing with 2 to 6 aliases per gene within and across species (Table 1 in *Theofanopoulou et al.*^2^), and this is causing confusion in the field and even with simple google searchers of online information. For example, our renamed *VTR2A* appears with many aliases in the NCBI and Ensembl databases: *VT1* in birds, *AVPR2.2* in frogs, *V2C*, *V2bR2* or *V2L* in fishes, and *V2C* or *V2bR2* in sharks, among other names (Table 1 in^2^). The gene we renamed to *VTR2C* appears as *AVPR2* or *V2R* in mammals, *V2A(2)* or *avpr2a(a)* in fishes, etc (Table 1 in^2^). In online definitions curated by scientists, vasotocin is described as the homolog of oxytocin and vasopressin (in Wikipedia) as if there are three different homologous genes, but we and others have shown^2^ that vasotocin and vasopressin are the same gene. We believe our new revisions improve communication about the genes and provide unique gene terms by getting rid of multiple aliases. Further, propagating the non-evolution-based oxytocin and arginine vasopressin terminology to the receptors will break an evolutionary based nomenclature already present in many non-mammalian species. Thus, we argue that *McCarthy et al.*^1^ have understated the existing issues and overstated the potential negative disruption of the *Theofanopoulou et al.*^2^’s recommended changes.

**Supplementary Note 3**

***VTR2A* and *VTR2C*: which is most likely the oldest and why**

In their supplementary comment^1^, *McCarthy et al.* disagreed with us^2^ that our named *VTR2A* (their *AVPR2C*) is evolutionarily older than our named *VTR2C* (their *AVPR2(A)*). In addition, since the publication of our study^2^, a new study by *Ocampo Daza et al.* 2022^13^ was published on further characterization of these genes, where they agreed with and adopted all our nomenclature changes, with a single exception, that the A and C of VTR2 should also be switched back to the common suffix used previously.

Our study, for the A to C suffix change was based on synteny results and species’ phylogeny^2^, where the renamed *VTR2A* and the genes surrounding it were inferred to be present in species near the origin of vertebrates, while the renamed *VTR2C* and its surrounding genes were inferred to be first present in the common ancestor of other vertebrates with bony fishes that appeared over 100 million years later. We found our renamed *VTR2A* and a set of syntenic genes in all vertebrates except coelacanths and mammals, and renamed *VTR2C* and its syntenic genes in all vertebrates except hagfishes, lampreys, sharks and birds. We trusted the synteny findings, as we analyzed synteny at multiple sequence length scales, including chromosomal, across 35 species, including high-quality VGP genomes, where we found no gaps or assembly errors in representative species that could result in synteny artifacts. Since the publication of our study, additional high-quality, chromosome-level genomes generated by the VGP became available for the sea lamprey and the white shark, which we now studied (**Supplementary Tables 1** and **2** of this study) and corroborated our results —namely, *VTR2A* present in both genomes and *VTR2C* in neither. The only other explanation of the synteny findings was that our renamed *VTR2C* and a large syntenic block flanking this gene was lost multiple independent times in all three basal branches of the vertebrate family tree (in jawless fishes, lampreys, and sharks).

Our conclusion that *VTR2C* first evolved by a segmental duplication from *VTR2A* or *VTR2B* in the common ancestor of bony fish with other vertebrates was in contrast to our phylogenetic analyses of nucleotide exon sequence alignments, were *VTR2C* branched off first as a sister group to *VTR2A* and *VTR2B*. However, in support of the synteny results, the protein coding sequence tree had our *VTR2A* branching off first, followed by *VTR2B* and *VTR2C*. Given the greater stability of the synteny phylogeny findings, and the instability of sequence alignment phylogeny findings, we gave priority to the synteny findings to give an evolution-based nomenclature for *VTR2A*, *VTR2B,* and *VTR2C*. We felt that a change was necessary, given the many aliases for *VTR2A* and *VTR2C* as mentioned above (Table 1 in^2^).

Although *McCarthy et al.* recognize *VTR2C*’s absence in the species we mentioned, they rely primarily on amino acid sequence phylogenetic results, which, like our nucleotide tree, suggest that *VTR2C* was older than *VTR2A* (their Supplementary Figure 1 in^1^); despite the similar conclusion, their tree topology was different (cascading relationships of the genes). According to their interpretation, this tree shows that “*the AVPR2 [VTR2C] gene first diverged from the common ancestor of the AVPR2C [VTR2A] and AVPR2B [VTR2B] genes prior to the duplication that gave rise to the AVPR2C [VTR2A] and AVPR2B[VTR2B]*” (our nomenclature is mentioned in []). Based on these findings, they favor the hypothesis that *AVP2R(A)* (our *VTR2C*) “*may have been present in the common ancestor of vertebrates and was subsequently lost in some lineages, including sharks*”.

*McCarthy et al.*^1^ generated their amino acid phylogeny only for the VTR2 genes, using sequences from 8 vertebrate species representing 5 lineages, yielding low bootstrap support (59-61%) on some of the key branches under discussion. Our amino acid phylogeny was based on sequences from c. 100 vertebrate species from the Ensembl database (rooted with the *Ciona*) (see Methods, Figure 4b in^2^), yielding >93% bootstrap for the key VTR2 relationships, but low support (~30-50%) for the VTR1 relationships. To shed further light on this issue, we ran a new alignment and phylogenetic tree using exonic nucleotide sequences coming mainly from chromosome-scale, high-quality assemblies, most of them generated by the VGP, to ensure the highest possible sequence quality and gene completeness. Our tree included OTR-VTR exonic sequences from 17 species representing 10 vertebrate lineages: 1 cyclostome: sea lamprey; 2 sharks: elephant shark and white shark, 1 coelacanth: coelacanth; 1 holost fish: spotted gar; 4 teleost fishes: zebrafish, red bellied piranha, electric eel, and blunt-snouted clingfish; 2 squamata: common wall lizard and Western terrestrial garter snake; 1 turtle: green sea turtle; 1 frog: tropical clawed frog; 2 birds: zebra finch and chicken; and 2 mammals: human and mouse (**Supplementary Table 1** for the accession and GenBank IDs of all the genomes used; **Supplementary Table 2**). The tree was rooted with the *VTR* sequence we identified in amphioxus^2^. We aligned the sequences with MAFFT (<https://mafft.cbrc.jp/>^8^; default parameters), and generated a Phylogenetic Maximum Likelihood tree using IQTree WebServer^9^ (1000 replicates), which we visualized via <https://phylo.io/>. All sequences used, FASTA alignment and Newick files can be accessed here: <https://github.com/constantinatheo/universalnomenclature/>.

Our updated phylogenetic tree reveals the same basic topology we had earlier, but now with strong bootstrap support (90-100%) on all major nodes (**Fig. 1**). *VTR2A* and *VTR2B* grouped together in a single node with clear confidence (99% support) and both branched with *VTR2C* (100%)*.* The low support values in *McCarthy et al.*’s tree^1^ can be due to: 1) fewer species and vertebrate lineages; 2) lower quality genome assemblies; and 3) only VTR2-sequences, instead of all OTR-VTR sequences in the phylogenies, suggestive that phylogenies on specific branches might not be able to yield as robust results; 4) using amino acid sequence instead of exonic nucleotide sequence, where the latter can yield more phylogenetic information (e.g.,^10^); and 5) rooting the phylogeny with the human *VTR1A* (their named *AVPR1A*), with other human VTR2-sequences used as ingroups. The taxa selection of the outgroup is crucial, since the outgroup method assumes that the outgroup-taxa sequence is divergent from the ingroup-taxa^11^. Our conclusion is that our updated tree more solidly points to a sister relationship of *VTR2A*/*VTR2B* on the one hand with *VTR2C* on the other, confirming our published exonic tree^2^. However, this more strongly reveals a conflict between the synteny-based results and the sequence alignment phylogeny results.

*Ocampo Daza et al.*^13^ analyzed additional genomes, including the shark and other cartilaginous fish genomes, and confirm the absence of *VTR2C* in them (whale shark, brownbanded bambooshark. cloudy catshark, and thorny skate; the latter generated by the VGP). However, they believe the absence is mainly due to assembly quality of cartilaginous fish genomes, which have more repetitive sequences, with the assumption that *VTR2C* is part of a hard to assemble multigenic region of the genome, although they provide no evidence that this is the case. We believe that *VTR2C* is simply not biologically present in cartilaginous fish genomes, as we could not even find evidence of the syntenic genes in shark and skate genomes.

*Ocampo Daza et al.*^13^ further argued that there is greater support for 2 rounds of whole genome duplication (2R-WGD) at the origin of vertebrates instead the model that *Theofanopoulou et al.*^2^ leaned towards of 1R-WGD, although *Theofanopoulou et al.*^2^ did not commit to either. *Ocampo Daza et al.*^13^ claim that we incorrectly identified several syntenic genes between paralogous chromosomes 1, 3, 7, 12, and X (human names), and that their synteny is more supportive of a 2R-WGD model, where eight *VTR genes* were generated nearly simultaneously, and then one from each subfamily (i.e. a hypothesized *VTR1C* and *VTR2D* in *Theofanopoulou et al.*^2^) was lost soon after the WGD events. According to their interpretation, *VTR2C* either was also lost multiple times or not assembled easily in species representing the basal lineages. Because of these contradictory findings, and still lack of complete resolution in sequence phylogeny and of complete genomes of the jawless and cartilaginous fishes, they strongly suggest interchanging the A and C suffixes of the VTR2 genes back to the commonly used designations.

We resonate with these concerns of a need to further resolve the cause of the conflicts in the evolutionary evidence and would not be against switching the *VTR2C* and *VTR2A* terminology to the commonly used A and C designations. If future evidence resolves the conflicts and more strongly supports an C and A swap, then we think that it should be followed. We note that the synteny results and sequence phylogeny results are consistent for all the other gene name revisions we made.

**Supplementary Note 4**

**Is *VTR2Ab* in teleost fish a *VTR2A* duplicate?**

In their supplementary comment^1^, *McCarthy et al.* also raise a point on our proposed orthologous relationship and nomenclature on the teleost fish-specific *VTR2Ab*^2^ (their named *avpr2l*)*,* an additional copy of *VTR2A*. Our conclusion was grounded on finding that this gene in teleost fish was located in the same chromosome with the first, *VTR2A* (*VTR2Aa* in teleost fish), as is the case for the rest of the teleost-specific copies of this gene family (*OTRb*, *VTR1Ab*, etc.; Supplementary Table 4b-e^2^). As *McCarthy et al.* note, we had not included this gene (*VTR2Ab*) in our exonic tree, which was due to its exonic nucleotide sequence at that time being too short, likely the result of an incomplete genome assembly^2^. But we had included it in our amino acid Ensembl tree, whose ID we provided, although we had collapsed its branch in Figure 4b^2^ for better visualization. We provide here the ID for the same tree focused on the branch under question ([ENSGT01020000230369](http://www.ensembl.org/Danio_rerio/Gene/Compara_Tree?db=core;g=ENSDARG00000076690;r=4:17836098-17838179;t=ENSDART00000146931)), based on the respective zebrafish gene (ID: ENSDARG00000076690), which shows that the proposed teleost-fish *VTR2Ab* branched directly with sea lamprey VTR2-sequences (*VTR2A* and *VTR2B*) with very low bootstrap support.

In the *McCarthy et al.* amino acid tree (Supplementary Figure 1^1^), *VTR2Ab* (their named *avpr2l*) sequences from two teleost fishes (zebrafish and southern platyfish) both branch outside of *VTR2C* (their named (*AVPR2(A)*) with low support (69%). This led *McCarthy et al.* to consider its identity uncertain, despite our synteny results, and not name it after any specific VTR2-type (*VTR2A*, *VTR2B* or *VTR2C*), but leave it as *avpr2l* (arginine vasopressin receptor 2-like).

In our new phylogenetic tree with exonic nucleotide OTR-VTR sequences mostly from the high-quality VGP genomes (**Fig. 1**), the electric eel *VTR2Ab* and zebrafish *VTR2Ab* branch with 100% support with the clingfish *VTR2Aa*, which is indicative of the former gene having evolved as a lineage-specific duplication of the latter. These teleost fish sequences form a 100% supported node with the white shark *VTR2A*, lending further evidence to the type of the receptor (*VTR2A*) that the gene under question (*VTR2Ab*) most likely belongs to. From there, this shark-teleost fish *VTR2A* node branched with the sea lamprey *VTR2A/B* genes at 96%, and altogether they branched with all other vertebrate *VTR2A* sequences at 80%. As shown in our published study (Supplementary Table 13 in^2^), the branching of both sea lamprey *VTR2A/B* in the *VTR2A* node can be explained due to their high GC content convergence.

This is a good example of a case for prioritizing synteny and combining it with phylogeny, whenever highly supported. Based on our synteny and the highly supported nodes from our phylogeny, teleost *VTR2Ab* is unequivocally most closely related to a *VTR2A*-receptor type, and not to a *VTR2B* or *VTR2C* receptor. Although the 80% support value linking the lamprey-shark-teleost fish *VTR2A* to the *VTR2A* in the rest of the vertebrates is not as high, it is comparable with the values we see in the rest of the clades for teleost-specific duplications (**Fig. 1**). In all these cases, synteny is the backbone of our identification of orthologous and paralogous relationships.

**Supplementary Note 5**

**Suggested checklist for evolutionary based gene nomenclatures.**

One of the issues that will keep coming up when scientists wish to propose a new gene nomenclature is the type of evidence needed to support such proposals. To our knowledge, a specific checklist of evidence has not been so far published. Although proposing a strict checklist of prerequisites falls out of the scope of this paper, we thought it would be useful to share the practices that we followed in *Theofanopoulou et al.*^2^, so that future studies can use it as a roadmap. Our checklist in the following order of priority is:

1. Synteny
2. Sequence alignment phylogeny
3. Sequence identity
4. Gene function

Further, considering gene synteny and sequence phylogeny, we have recommendations for the type of data quality and parameters used (**Extended Data Fig. 1**).

For synteny analyses, we used, to the extent possible, high-quality and chromosome-scale genomes from species representing all vertebrate major classes. We compared clusters of conserved genes around the genes of interest in different windows (from a 10-gene window to a 100-gene window, and a chromosomal-window), a practice we suggest is helpful, especially since synteny starts to break up and become less robust the more distantly related the compared species. An additional caveat to consider^6^ is that gene name annotations surrounding the gene of interest might themselves be erroneous, something requiring further attention. Divergence time needs to be considered upon deciding the different thresholds and parameters. For instance, in our chromosomal-window synteny analyses^2^ in closely related species, we set that at least 3 homologous genes should be found in a 20-gene distance for them to be considered syntenic.

For phylogenetic inferences, we suggest that the same genome quality and number of species represented should hold. We also suggest that at least three inferences should be made, trees based on: protein coding sequences; exonic sequences; and full-length sequences. As noted in **Supplementary Note 3**, DNA sequence-based trees have been found to yield more phylogenetic information than those based on protein sequences (e.g.,^10^). Additionally, the trees should be rooted with an outgroup-taxa sequence. There are also some caveats when it comes to understanding evolutionary relationships based on tree inferences only. For example, inferences on genes with short sequences tend to be less reliable than inferences on genes with long sequences^12^. Additionally, it is difficult to decide on a specific bootstrap cut-off (e.g., values ≥80%), since specific questions can be answered with different thresholds of support. For example, in our findings on the *VTR2B* branch (**Fig. 1**), the zebrafish and eel *VTR2Ba* sequences branch with the spotted gar, coelacanth and clingfish sequences with 73% support, while all these sequences together branch with the piranha *VTR2Ba* with 99% support. If we had a strict ≥80% threshold, and if synteny results were not clear in this case, the identity of these zebrafish and eel sequences would be seemingly obscure. But in this specific instance, we know that often teleost fish sequences tend to have high divergence rates, and that lower support values (e.g., 73%) are expected in a highly supported branch (e.g., 99%) of orthologs. In other words, accuracy in phylogenetic inferences cannot be easily defined based on absolute bootstrap values alone. Multiple factors should be taken into account for each gene family. If none of these criteria (synteny and phylogeny) provide useful information for a nomenclature decision, we do not think that such decision should be made on the basis of sequence identity or gene function alone.

**References**

1. McCarthy, Fiona M., Jones, Tamsin E.M., Kwitek, Anne E., Smith, Cynthia L., Vize, Peter D., Westerfield, Monte, Bruford, E. A. The Case for Standardising Gene Nomenclature across Vertebrates.

2. Theofanopoulou, C., Gedman, G., Cahill, J. A., Boeckx, C. & Jarvis, E. D. Universal nomenclature for oxytocin–vasotocin ligand and receptor families. *Nature* **592**, 747–755 (2021).

3. Kent, W. J. BLAT---The BLAST-Like Alignment Tool. *Genome Res.* **12**, 656–664 (2002).

4. Altschul, S. F., Gish, W., Miller, W., Myers, E. W., & Lipman, D. J. Basic local alignment search tool. *J. Mol. Biol.* **215**, 403–410 (1990).

5. Lyons, E. & Freeling, M. How to usefully compare homologous plant genes and chromosomes as DNA sequences. *Plant J.* **53**, 661–673 (2008).

6. Haug-Baltzell, A., Stephens, S. A., Davey, S., Scheidegger, C. E. & Lyons, E. SynMap2 and SynMap3D: Web-based whole-genome synteny browsers. *Bioinformatics* **33**, 2197–2198 (2017).

7. Rhie+, Arang, McCarthy+, Shane, Fedrigo, Olivier, VGP Flagship Assembly Paper Authors, Howe, Kerstin, Myers, Eugene W., Durbin, Richard, Phillippy, Adam M., Jarvis, E. D. Towards complete and error-free genome assemblies of all vertebrate species. *Nature* (2021).

8. Nakamura, T., Yamada, K. D., Tomii, K. & Katoh, K. Parallelization of MAFFT for large-scale multiple sequence alignments. *Bioinformatics* **34**, 2490–2492 (2018).

9. Trifinopoulos, J., Nguyen, L.-T., von Haeseler, A. & Minh, B. Q. W-IQ-TREE: a fast online phylogenetic tool for maximum likelihood analysis. *Nucleic Acids Res.* **44**, W232–W235 (2016).

10. Hall, B. G. Comparison of the Accuracies of Several Phylogenetic Methods Using Protein and DNA Sequences. *Mol. Biol. Evol.* **22**, 792–802 (2005).

11. Kinene, T., Wainaina, J., Maina, S. & Boykin, L. M. Rooting Trees, Methods for. in *Encyclopedia of Evolutionary Biology* 489–493 (Elsevier, 2016). doi:10.1016/B978-0-12-800049-6.00215-8.

12. Jarvis, E. D. *et al.* Whole-genome analyses resolve early branches in the tree of life of modern birds. *Science (80-. ).* **346**, 1320–1331 (2014).

13. Ocampo Daza, D., Bergqvist, C. A. & Larhammar, D. The Evolution of Oxytocin and Vasotocin Receptor Genes in Jawed Vertebrates: A Clear Case for Gene Duplications Through Ancestral Whole-Genome Duplications. *Front. Endocrinol. (Lausanne).* **12**, (2022).
