# Supplementary material for: Reply to: The case for standardizing gene nomenclature in vertebrates
Source: Nature. 2023 Feb 15;614(7948):E33–6. doi: 10.1038/s41586-022-05634-9 (PMC9931572; doi:10.1038/s41586-022-05634-9)
Supplement: Supplementary file 2 — Reporting Summary [file 41586_2022_5634_MOESM2_ESM.pdf]

nature research

Corresponding author(s): Theofanopoulou C.; Jarvis E.D.

Last updated by author(s): October 27th, 2022

Reporting Summary

Nature Research wishes to improve the reproducibility of the work that we publish. This form provides structure for consistency and transparency in reporting. For further information on Nature Research policies, see Authors & Referees and the Editorial Policy Checklist.

Statistics

For all statistical analyses, confirm that the following items are present in the figure legend, table legend, main text, or Methods section.

n/a	Confirmed	
☒	☐	The exact sample size (n) for each experimental group/condition, given as a discrete number and unit of measurement
☒	☐	A statement on whether measurements were taken from distinct samples or whether the same sample was measured repeatedly
☒	☐	The statistical test(s) used AND whether they are one- or two-sided<br>*Only common tests should be described solely by name; describe more complex techniques*
☒	☐	A description of all covariates tested
☒	☐	A description of any assumptions or corrections, such as tests of normality and adjustment for multiple comparisons
☒	☐	A full description of the statistical parameters including central tendency (e.g. means) or other basic estimates (e.g. regression coefficient) AND variation (e.g. standard deviation) or associated estimates of uncertainty (e.g. confidence intervals)
☒	☐	For null hypothesis testing, the test statistic (e.g. F, t, r) with confidence intervals, effect sizes, degrees of freedom and P value noted<br>*Give P values as exact values whenever suitable.*
☒	☐	For Bayesian analysis, information on the choice of priors and Markov chain Monte Carlo settings
☒	☐	For hierarchical and complex designs, identification of the appropriate level for tests and full reporting of outcomes
☒	☐	Estimates of effect sizes (e.g. Cohen's d, Pearson's r), indicating how they were calculated

Our web collection on statistics for biologists contains articles on many of the points above.

Software and code

Policy information about availability of computer code

Data collection	We collected our data using gene sequences found in the NCBI (https://www.ncbi.nlm.nih.gov/) and Ensembl (release 105; https://useast.ensembl.org/index.html). Genome assembly IDs and GenBank assembly accession numbers can be found in Supplementary Table 1.
Data analysis	Our exonic sequences were aligned with MAFFT (v 7) (https://mafft.cbrc.jp/8; default parameters); from this alignment, we generated a Phylogenetic Maximum Likelihood tree using IQTree WebServer (1000 replicates) (v2.2.0), which we visualized via https://phylo.io/.

For manuscripts utilizing custom algorithms or software that are central to the research but not yet described in published literature, software must be made available to editors/reviewers. We strongly encourage code deposition in a community repository (e.g. GitHub). See the Nature Research guidelines for submitting code & software for further information.

Data

Policy information about availability of data

All manuscripts must include a data availability statement. This statement should provide the following information, where applicable:

- Accession codes, unique identifiers, or web links for publicly available datasets
- A list of figures that have associated raw data
- A description of any restrictions on data availability

We used 17 vertebrate and 1 invertebrate species' genomes, whose IDs and GenBank assembly accession numbers can be found in Supplementary Table 1. All the NCBI/Ensembl/Gene IDs of the genomes and genes we included in the phylogenetic tree can be found in the Suppl.Tables_TheofanopoulouMattersArising excel document All the exonic gene sequences, alignment and Newick tree files used for the phylogenetic tree can be found here: https:// github.com/constantinatheo/universalnomenclature.

Field-specific reporting

Please select the one below that is the best fit for your research. If you are not sure, read the appropriate sections before making your selection.

☒ Life sciences ☐ Behavioural & social sciences ☐ Ecological, evolutionary & environmental sciences

For a reference copy of the document with all sections, see nature.com/documents/nr-reporting-summary-flat.pdf

Life sciences study design

All studies must disclose on these points even when the disclosure is negative.

Sample size

For the exonic phylogeny we used the longest read-sequences available from species representing 10 vertebrate lineages (1 cyclostome: sea lamprey; 2 sharks: elephant shark and white shark, 1 coelacanth: coelacanth; 1 holost fish: spotted gar; 4 teleost fishes: zebrafish, red bellied piranha, electric eel, and blunt-snouted clingfish; 2 squamata: common wall lizard and Western terrestrial garter snake; 1 turtle: green sea turtle; 1 frog: tropical clawed frog; 2 birds: zebra finch and chicken; and 2 mammals: human and mouse) and 1 invertebrate (amphioxus). No sample size calculation was performed; sample size was determined sufficient in terms of quantity, since all major vertebrate lineages are represented, and quality, since we used high-quality genomes wherever available.

Data exclusions

We did not exclude any genomes of species that would have contributed further to the understanding of the evolution of the OTR-VTR receptors.

Replication

We generated a Phylogenetic Maximum Likelihood tree using IQTree WebServer (1000 replicates). All attempts for replication were successful.

Blinding

Our tests were blind in that we had not assigned specific names to the genes before our synteny analyses showed clearly which gene is orthologous to which (Theofanopoulou et al. 2021).

Randomization

Randomization was not relevant in this study, since it is impossible to randomize the gene sequences found in a species' genome.

Reporting for specific materials, systems and methods

We require information from authors about some types of materials, experimental systems and methods used in many studies. Here, indicate whether each material, system or method listed is relevant to your study. If you are not sure if a list item applies to your research, read the appropriate section before selecting a response.

Materials & experimental systems

n/a	Involved in the study	
☒	☐	Antibodies
☒	☐	Eukaryotic cell lines
☒	☐	Palaeontology
☒	☐	Animals and other organisms
☒	☐	Human research participants
☒	☐	Clinical data

Methods

n/a	Involved in the study	
☒	☐	ChIP-seq
☒	☐	Flow cytometry
☒	☐	MRI-based neuroimaging
